# The influence of Javanese turmeric (*Curcuma xanthorrhiza*) on the pharmacokinetics of warfarin in rats with single and multiple-dose studies

**DOI:** 10.1080/13880209.2021.1928716

**Published:** 2021-06-01

**Authors:** Taofik Rusdiana, Yanni D. Mardhiani, Norisca A. Putriana, Dolih Gozali, Daisuke Nagano, Takuya Araki, Koujirou Yamamoto

**Affiliations:** a Department of Pharmaceutics and Pharmaceutical Technology, Faculty of Pharmacy, Universitas Padjadjaran, Sumedang, Indonesia; b Faculty of Pharmacy, Bhakti Kencana University, Bandung, Indonesia; c Department of Clinical Pharmacology and Therapeutics, Graduate School of Medicine, Gunma University, Maebashi, Japan

**Keywords:** AUC, biological half-life, drug-herb interaction

## Abstract

**Context:**

Co-administration between warfarin (WF) and *Curcuma xanthorrhiza* Roxb. (Zingiberaceae) (CX) is found in Indonesian patients and need to be evaluated.

**Objective:**

This study assesses the effect of concomitant administration of CX extract on the pharmacokinetics of WF in rats.

**Materials and methods:**

Wistar rats were divided into 4 groups (*n* = 6) and administered with 2% Pulvis Gummi Arabicum (PGA, control), fluconazole (FZ, 6 mg/kg), CX-1 (6 mg/kg) or CX-2 (18 mg/kg BW) for 7 days. For the single-dose study, at the 8th day, WF (1 mg/kg) was administered to all groups and blood samples were taken from 0.25 to 72 h. For the multiple-dose study, daily dose of WF was administered to all groups of rats and at the 7th to 9th day, the rats were treated with PGA, CX-1, CX-2 and FZ. Blood samples were withdrawn daily at 4 h after administration of WF from the 1st to 11th day.

**Results:**

The area under the curve (AUC) of *R*- and *S*-WF in the CX-2 group was a significantly higher value compared to the control (77.54 vs. 35.27 mg.h/L for *R*-WF and 316.26 vs. 40.16 mg.h/L for *S*-WF; *p* < 0.05; Kruskal-Wallis method). The CX-2 administration also caused the increasing in the concentration level of *R*-WF (16%) and *S*-WF (27%) from the 7th to 9th day of administration.

**Discussion and Conclusions:**

The CX administration in a higher dose caused alteration on WF pharmacokinetics suggesting the need for clinical evaluation of the interaction between CX and WF.

## Introduction

Warfarin (WF) is still widely used as an anticoagulant drug for prevention and treatment of the diseases associated with thromboembolism such as atrial fibrillation, deep vein thrombosis, and pulmonary thromboembolism (Hirsh et al. 2001). It has two active enantiomers which are *R-* and *S*-form. The *S* is 3–5 times more potent than the other form in terms of its anticoagulant activity. However, in the body, the *S* is eliminated faster than the *R* (Kaminsky and Zhang 1997; Rusdiana et al. 2013). The main problem in warfarin therapy is a high inter-individual variation in patients’ response with regards to both the pharmacokinetics and pharmacodynamics, which lead to the adverse drug reaction such as bleeding events and thromboembolism complication. It is well known that WF has a narrow therapeutic index which causes its therapy to easily fall into an over or under dose. This situation is increasingly difficult to control due to genetic polymorphisms in the metabolizing enzymes and receptor of WF (Rusdiana et al. 2013). Significant variability in warfarin response is caused by patient demographics (age, weight, sex, body surface area) (Yoo et al. 2009; Khoury and Sheikh-Taha 2014; Marcatto et al. 2016; Bernaitis et al. 2017), diets (Ovesen et al. 1988; Custodio das Dores et al. 2007; Haffa et al. 2010; Rasmussen et al. 2012; Sasaki et al. 2012), smoking behaviours (Mitchell 1972; Bachmann et al. 1979; Nathisuwan et al. 2011), genetic factors of VKORC1, CYP2C9 and CYP4F2 (Mushiroda et al. 2006; Obayashi et al. 2006; Yoshizawa et al. 2009; Kato et al. 2011; Miyagata et al. 2011; Tanaka et al. 2013; Wakamiya et al. 2016) plus interactions with drugs, foods and herbs (Kong et al. 1995; Zhu et al. 1999; Makino et al. 2002; Mohammed Abdul et al. 2008; Ansell et al. 2009; Panossian et al. 2009; Zhou et al. 2012; Yan et al. 2014; Lagishetty et al. 2016; Li et al. 2016; Shen et al. 2016; Ramanathan and Penzak 2017; Ueng et al. 2017; Zhao et al. 2017; Guo et al. 2018; Vranckx et al. 2018; Werba et al. 2018; Zhang et al. 2018).

Several studies on pharmacokinetic interaction of WF and herbal medicine showed an alteration in the parameters such as elimination half-life (t_1/2_), area under the curve (AUC), volume of distribution (V) and clearance (C_L_). For instance, an administration of andrographolide before the treatment of warfarin exhibited increased C_max_ and AUC as well as t_1/2_ of WF (Zhang et al. 2018). Werba et al. (2018) reported the effect of green tea which influenced the WF pharmacodynamics, that the tea reduced the value of INR (International Normalized Ratio) significantly from 3.79 to 1.37. Also, the high vitamin K content in it is associated with the mechanism of decreasing WF effect (Cheng 2007). But other studies revealed that some herbs stimulate an induction or inhibition on metabolizing enzymes of cytochrome P450 or modulating the activity of P-gp transporter such as aloe, jalap, cascara, and rhubarb (Li et al. 2016). Consequently, these tend to influence the pharmacokinetics of warfarin.

*Curcuma xanthorrhiza* Roxb. (CX), a member of the ginger family (Zingiberaceae), known as Javanese turmeric, is a common herb used in *Jamu* (Indonesian traditional medicine) prescription or other health supplements (Widyowati and Agil 2018). Main compounds of CX are curcuminoids and xanthorrhizol oil which have antioxidant, anti-inflammation, anticancer, hepatoprotective, and antibacterial activities (Itokawa et al. 2008; Rukayadi and Hwang 2013; Ismail et al. 2017). Other findings reported that the major compounds were xanthorrhizol, champene, and α-curcumene (Widyowati and Agil 2018) which have potential effects to cause significant herb-drug interaction that are primarily metabolized by CYP3A4/A5, CYP2C9, and UGT enzymes (Volak et al. 2008; Salleh et al. 2016). Since warfarin is known as one of the drugs dominantly metabolized by CYP2C9 (for *S*-WF) and CYP3A4 (for *R*-WF), it could be hypothesized that CX extract influences or alters the pharmacokinetics of WF. The case of a coadministration between WF and CX is common in the patients with cardiovascular related disease because of self-medication or prescription. This means, many subjected to WF therapy are also good consumers of the extract for health maintenance. Therefore, this study aims to assess the influence of CX extract administration on the pharmacokinetic of WF in rats through single and multiple-dose.

## Materials and methods

### Materials

The materials used were warfarin USP Reference standard and ethyl carbamate (Wako, PT. Nebelin, Indonesia), WF pharmaceutical grade (PT. Fahrenheit, Indonesia), *Curcuma xanthorrhiza* extract (PT. Konimex, Indonesia), Fluconazole (Sigma Aldrich, Japan), Pulvis Gom Arabicum, PGA (PT. Bratachem, Bandung, Indonesia), heparin (Wako, Japan), 1.5 mL anticoagulant-containing tube, SP10 and SP31 tubing, 23 G needle, 1 mL and 3 mL syringe, string and one set of surgery tools.

### Ethical clearance

This study and all protocols were ethically approved by The Research Ethics Committee of Universitas Padjadjaran Hospital, Bandung, Indonesia (No. 1365/UN6.KEP/EC/2018).

### Animal and study design

#### Single-dose study

Twenty-four male Wistar rats were used, and divided into 4 groups (Control, FZ, CX-1, and CX-2), weighed around 250–300 g, maintained and housed with a 12 h light/dark cycle at room temperature (about 25 °C). One week before warfarin was given, all rats in each group were subjected to daily pre-treatment by oral administration of *Curcuma xanthorrhiza* extract at the usual dose (6 mg/kg BW, grouped as CX-1) and at a high dose (3 times the usual, 18 mg/kg BW, grouped as CX-2), Fluconazole at a dose of 6 mg/kg BW (grouped as FZ) and 2% PGA as a control. After 7 days, they were then administered WF intravenously at a dose of 1 mg/kg BW via the femoral vein. Note that the usual dose of CX extract used in this experiment was calculated based on the adult human daily dose administration of 60–120 mg (Ikatan Apoteker Indonesia [IAI] 2017).

#### Multiple-dose study

Twenty-four male Wistar rats were also used, which were divided into 4 groups (control, CX-1, CX-2 and FZ). All of them were treated with WF at a dose of 0.2 mg/kg BW from the 1st to 11th day. For three days, which was on 7th to 9th, the rats were treated with 2% PGA for the control group, 6 mg/kg of the extract for CX-1, and 18 mg/kg of the extract for CX-2 and 6 mg/kg of Fluconazole for FZ. Blood samples were withdrawn every day at 4 h after administration of WF. The plasma was separated from each blood sample and stored at −80 °C for HPLC analysis of warfarin content.

### Blood sampling

#### Single-dose study

Blood samples (±0.5 mL) were withdrawn from the femoral artery at 15 min before drug treatment (blank plasma) and 0.25, 0.5, 1, 2, 4, 8, 12, 24, 36, 48, 60, and 72 h after WF administration and collected into 1.5 mL heparin-containing tube. Then, the plasma (±0.2 mL) was obtained by centrifugation at 2800 rpm for 15 min and stored at −20 °C until the measurement of WF enantiomers concentration.

#### Multiple-dose study

Blood samples (±0.5 mL) were withdrawn daily from on 1st to 11th at 4 h after the administration of warfarin. This time was determined based on the calculation of t_max_ (ss) which mean the time required for maximum concentration of drug to be in the steady-state condition [C_max_ (ss)]. Furthermore, the plasma was separated from every blood sample and stored at −80 °C until ready for HPLC assay of WF concentration.

### Determination of WF enantiomers’ concentration in rat plasma

The measurements of warfarin concentrations were performed by using the HPLC according to the method as previously described (Rusdiana et al. 2013). A Chiralcel OD-RH column (4.6 mm × 150 mm; Daicel Chemical Industries, Tokyo, Japan) was used and maintained at 40 °C. Moreover, the mobile phase was 20 mmol/L potassium phosphate buffer (pH 2.0)/acetonitrile (65/35 [v/v]), which was pumped at a flow rate of 1.0 mL/min. In sample preparation, 50 µL of 10 µg/mL indomethacin as an internal standard (IS) and 0.1 mL of 2 mol/L hydrochloric acid were added to 0.1 mL of the plasma. After vortex-mixing, 0.5 mL of ethyl acetate was added and the mixture was shaken for 30 min followed by centrifugation at 13,000 rpm for 4 min. Then, the upper organic phase was transferred and evaporated to dryness under reduced pressure at room temperature (under 1.3 kPa at 20–25 °C). Finally, the residue was reconstituted with 100 µL of the mobile phase, and 80 µL of the solution was injected into the HPLC system.

### Calculation of pharmacokinetic parameters

After obtaining *R*- and *S*-WF level in the blood samples, a pharmacokinetic profile of plasma concentration versus time in each rat was plotted. Also, the pharmacokinetic model and parameters including elimination rate constant (k), biological t_1/2_, AUC, the volume of distribution (Vd), and clearance (C_L_) were determined using the Excel MS-office 2013 and verified by PK solver application.

### Statistical analysis

A difference between the pharmacokinetic parameters of *R*- and *S*-WF in the control and treatment groups was analysed through the Kruskal-Wallis or Man-U test method, with a level of significance at α = 0.05 by using the R-commander (a graphical user interface).

## Results

### Single-dose study

**Figure 1.**
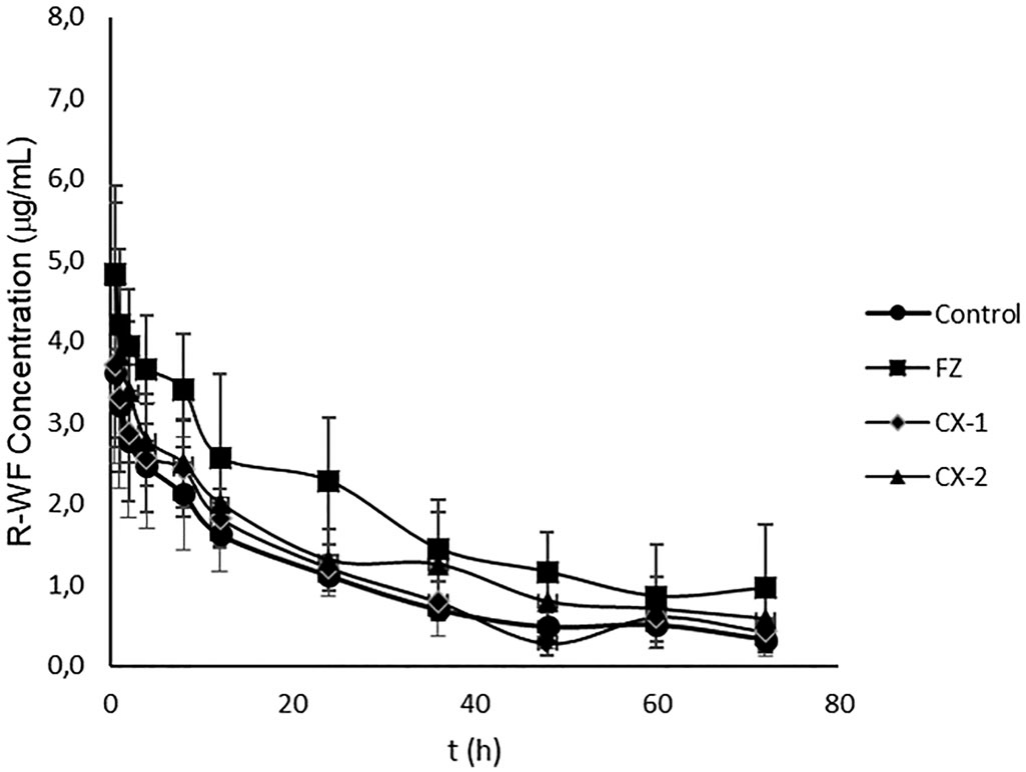
Plasma concentration curve of R-WF in control and treatment groups; closed cycle: control group-Pulvis Gum Arabicum (2% PGA); closed rectangle: FZ group – Fluconazole at a dose of 6 mg/kg BW. Closed diamond: CX-1 – CX extract at a dose of 6 mg/kg BW; closed triangle: CX-2 is CX extract at a dose of 30 mg/kg BW.

**Figure 2.**
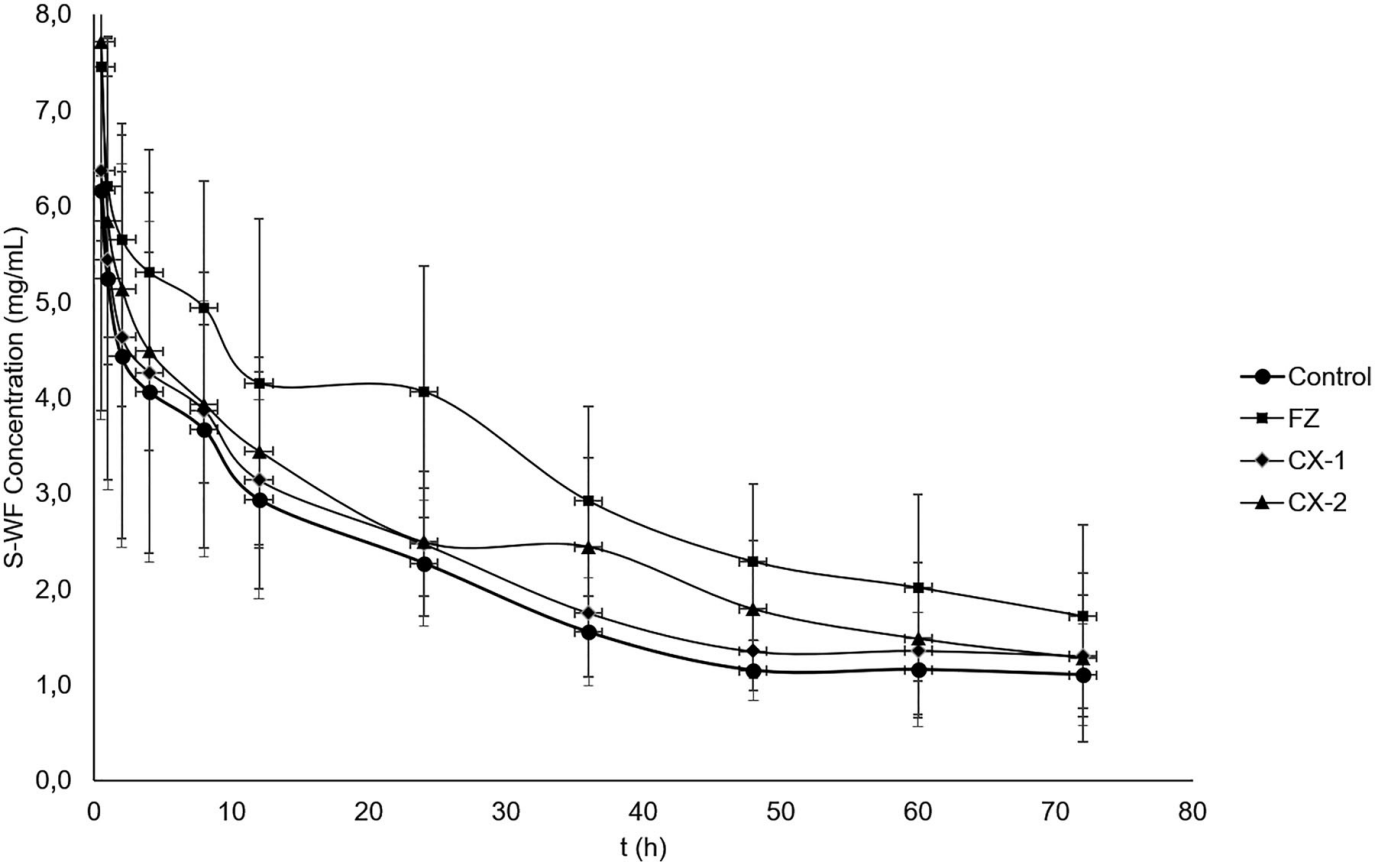
Plasma concentration curve of S-WF in control and treatment groups; closed cycle: control group-Pulvis Gum Arabicum (2% PGA); closed rectangle: FZ group – Fluconazole at a dose of 6 mg/kg BW. Closed diamond: CX-1 – CX extract at a dose of 6 mg/kg BW; closed triangle: CX-2 is CX extract at a dose of 30 mg/kg BW.

The concentration of *R*- and *S*-WF in the rat after administration and treatment with PGA (control), Fluconazole (positive control), and *Curcuma xanthorriza* extract (CX-1 and CX-2) for the single-dose study are summarized in Table 1. In addition, the profiles of plasma concentration curve for *R*- and *S*-WF in the four groups are presented in Figures 1 and 2. These showed that the curves in the CX-2 and FZ groups seem to be higher than in the control both for *R*-WF and *S*-WF. However, the curve in the CX-1 group seems to be closer to that of the control as seen in Figures 1 and 2. To make deep analysis, the pharmacokinetic parameters were further calculated including k (elimination constant rate), t_1/2_ (elimination half live), Vd, C_L_, AUC, MRT (mean residence time) and V_ss_ (volume of distribution in the steady-state condition) for the *R*- and *S-*WF in the four groups as summarized in Table 2.

**Table 1.** The concentration of *R*- and *S-*WF in rats after concomitant administration with CX extract.

t (h)	*R*-WF Concentration (µg/mL), *n* = 6				*S*-WF Concentration (µg/mL), *n* = 6			
	Control	FZ	CX-1	CX-2	Control	FZ	CX-1	CX-2
0.5	3.61 ± 1.10	4.83 ± 1.09	3.71 ± 1.00	4.81 ± 0.91	6.17 ± 2.40	7.45 ± 1.82	6.37 ± 2.50	7.71 ± 1.39
1	3.22 ± 1.03	4.21 ± 0.93	3.32 ± 0.93	3.83 ± 1.01	5.24 ± 2.20	6.20 ± 1.57	5.44 ± 2.30	5.85 ± 1.50
2	2.77 ± 0.94	3.94 ± 0.72	2.87 ± 0.84	3.38 ± 0.87	4.44 ± 2.00	5.65 ± 1.21	4.64 ± 2.10	5.14 ± 1.23
4	2.47 ± 0.77	3.66 ± 0.67	2.57 ± 0.67	2.79 ± 0.56	4.06 ± 1.78	5.31 ± 1.29	4.26 ± 1.88	4.49 ± 1.04
8	2.13 ± 0.69	3.40 ± 0.70	2.43 ± 0.59	2.51 ± 0.54	3.67 ± 1.34	4.94 ± 1.32	3.87 ± 1.44	3.94 ± 0.83
12	1.63 ± 0.46	2.58 ± 1.02	1.83 ± 0.36	2.01 ± 0.52	2.94 ± 1.04	4.15 ± 1.72	3.14 ± 1.14	3.44 ± 0.98
24	1.12 ± 0.25	2.28 ± 0.77	1.22 ± 0.15	1.31 ± 0.39	2.27 ± 0.65	4.06 ± 1.31	2.47 ± 0.75	2.49 ± 0.57
36	0.70 ± 0.34	1.45 ± 0.61	0.80 ± 0.24	1.26 ± 0.65	1.55 ± 0.56	2.92 ± 0.99	1.75 ± 0.66	2.44 ± 0.93
48	0.49 ± 0.26	1.16 ± 0.49	0.29 ± 0.16	0.80 ± 0.44	1.15 ± 0.31	2.29 ± 0.81	1.35 ± 0.41	1.80 ± 0.72
60	0.51 ± 0.29	0.86 ± 0.64	0.61 ± 0.19	0.71 ± 0.40	1.16 ± 0.60	2.02 ± 0.97	1.36 ± 0.70	1.49 ± 0.79
72	0.33 ± 0.22	0.96 ± 0.78	0.43 ± 0.12	0.59 ± 0.41	1.10 ± 0.53	1.72 ± 0.96	1.30 ± 0.63	1.29 ± 0.88

Control: 2% PGA; FZ: Fluconazole at a dose of 6 mg/kg BW. CX-1: CX extract at a dose of 6 mg/kg BW; CX-2: CX extract at a dose of 30 mg/kg BW. n: number sample (rat), values are presented as mean ± SD, SD: Standard Deviation.

**Table 2.** Pharmacokinetic parameters of *R*- and *S*-WF in rats after administration of CX extract concomitantly.

PK Parameter	Unit	*R*-WF				*S*-WF			
		Control	FZ	CX-1	CX-2	Control	FZ	CX-1	CX-2
k	1/h	0.07	0.05	0.05	0.04*	0.04	0.04	0.09	0.03*
t_1/2_	h	16.03	20.51	13.86	19.77*	28.71	28.50	16.21	74.26*
V	L	0.04	0.02	0.02	0.03	0.06	0.02	0.02	0.01*
C_L_ (*10^–3^)	L/h	3.00	1.00	2.00	1.00*	2.50	0.60	1.10	0.30*
AUC 0-t	mg.h/L	33.78	78.23**	51.78	71.36**	33.43	142.65**	89.28*	159.27**
AUC 0-inf	mg.h/L	35.27	85.71**	52.59	77.54**	40.16	172.17**	93.48*	316.26**
MRT	H	22.00	29.30	16.90	28.40	39.62	40.63	22.88	104.19*
Vss	L	0.06	0.03	0.03	0.04	0.10	0.02	0.02	0.03

Control: 2% PGA; FZ: Fluconazole at a dose of 6 mg/kg BW. CX-1: CX extract at a dose of 6 mg/kg BW; CX-2: CX extract at a dose of 30 mg/kg BW; k is elimination rate constant (sentral compartment); t_1/2_ is biological half-life; V is volume of distribution; CL is drug clearance; AUC_0-t_ is Area Under the Curve from zero to t last time of observation; AUC_0-inf_ is Area Under the Curve from zero to infinitive time (AUC total); MRT is Mean Residence Time; V_ss_ is volume of distribution in steady state.

*The difference is statistically significant at a level of *p* < 0.05; **The difference is statistically significant at a level of *p* < 0.01 (by Kruskall-Wallis method).

### Multiple-dose study

**Figure 3.**
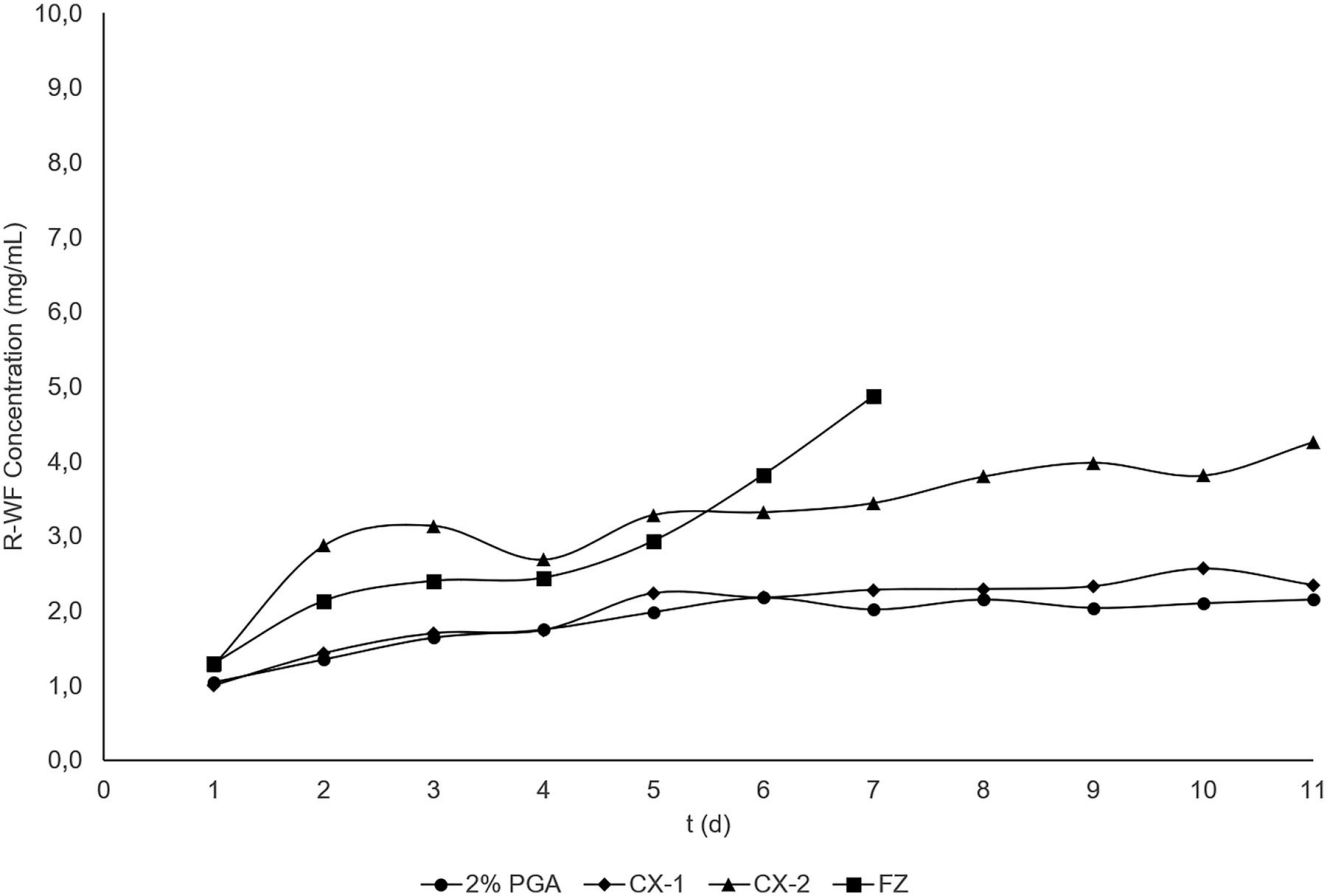
Plasma concentration curve of R-WF in control and treatment groups with multiple-dose administration; closed cycle: control group, Pulvis Gum Arabicum (2% PGA); closed rectangle: FZ group, Fluconazole at a dose of 6 mg/kg BW. Closed diamond: CX-1, CX extract at a dose of 6 mg/kg BW; closed triangle: CX-2, CX extract at a dose of 30 mg/kg BW. SD bar is not displayed in this figure.

**Figure 4.**
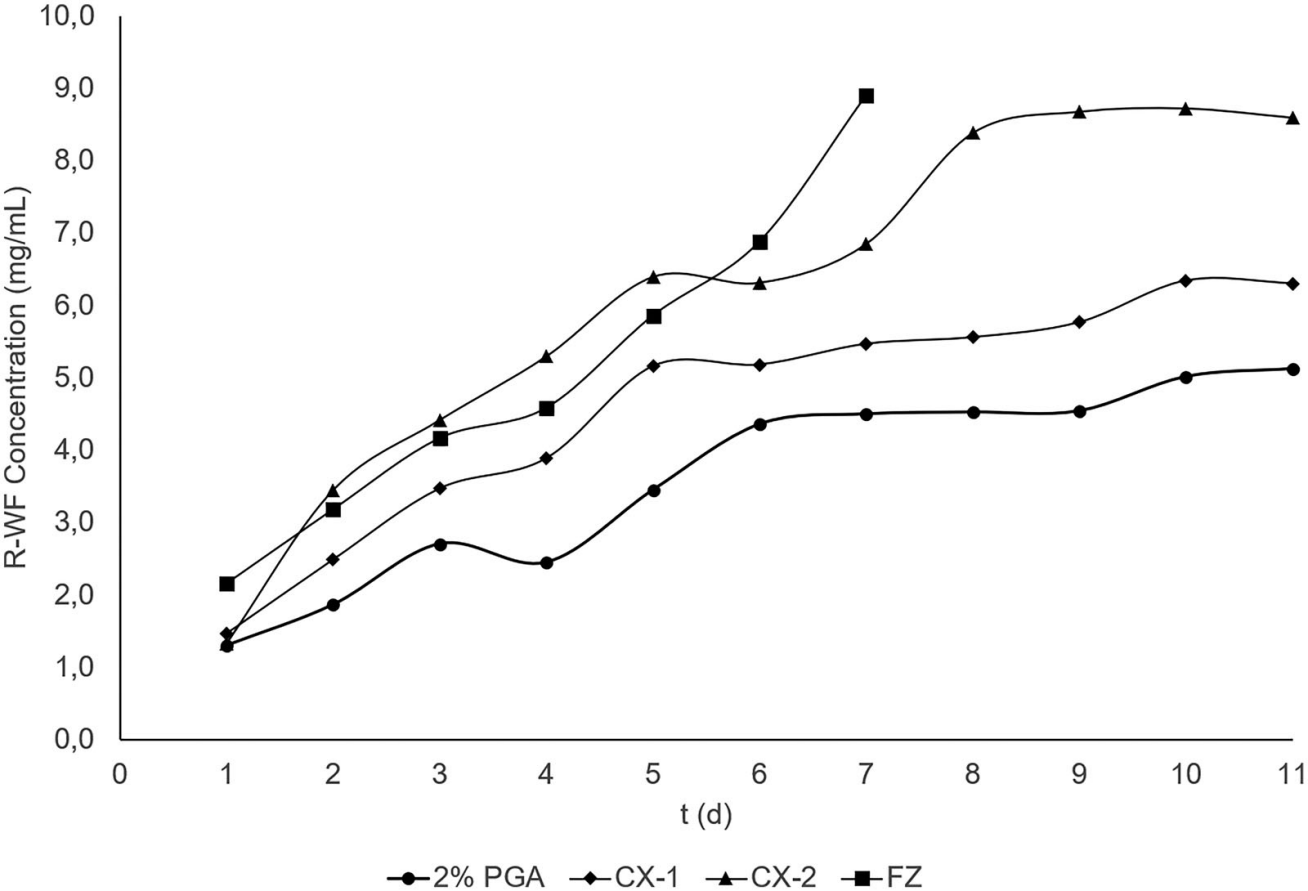
Plasma concentration curve of S-WF in control and treatment groups with multiple-dose administration; closed cycle: control group, Pulvis Gum Arabicum (2% PGA); closed rectangle: FZ group, Fluconazole at a dose of 6 mg/kg BW. Closed diamond: CX-1, CX extract at a dose of 6 mg/kg BW; closed triangle: CX-2, CX extract at a dose of 30 mg/kg BW. SD bar is not displayed in this figure.

The concentrations of *R*- and *S*-WF used are presented in Table 3 and the profiles of warfarin plasma curve are in Figure 3 for *R*-WF and 4 for *S*-WF. More also, the line curve of the WF concentration in CX-2 is much higher compared to the control. Similarly, the concentration of warfarin for the FZ group was higher than that of the control during the initial day of administration. The analysis was not furthered because of samples discontinuation, which is after the 8th day of observation where almost all rats in the FZ group had major bleeding and died. Based on Figures 3 and 4, the curve profiles in the CX-1 almost coincided with those of the control group. For further analysis, the difference of *R*- and *S*-WF concentration in each group was calculated from the 7th to 9th day (a period of administering the treatment of CX extract, FZ and control) as presented in Table 4.

**Table 3.** The concentration of *R*- and *S*-WF in the four groups of rats from day of 1–11 using multiple-dose study.

t (day)	Control (µg/mL)		CX-1 (µg/mL)		CX-2 (µg/mL)		FZ (µg/mL)	
	*R*-WF	*S*-WF	*R*-WF	*S*-WF	*R*-WF	*S*-WF	*R*-WF	*S*-WF
1	1.05	1.31	1.00	1.47	1.28	1.34	1.30	2.17
2	1.35	1.87	1.43	2.49	2.87	3.45	2.13	3.19
3	1.65	2.71	1.70	3.47	3.14	4.42	2.40	4.17
4	1.75	2.45	1.74	3.89	2.69	5.30	2.44	4.59
5	1.99	3.45	2.24	5.16	3.29	6.40	2.94	5.86
6	2.18	4.36	2.18	5.18	3.32	6.32	3.82	6.89
7	2.02	4.50	2.28	5.47	3.44	6.85	4.87	8.90
8	2.15	4.52	2.29	5.56	3.80	8.39	NA	NA
9	2.04	4.54	2.33	5.79	3.98	8.68	NA	NA
10	2.10	5.02	2.57	6.34	3.81	8.72	NA	NA
11	2.15	5.12	2.34	6.30	4.26	8.60	NA	NA

Control: 2% PGA; FZ: Fluconazole at a dose of 6 mg/kg BW. CX-1: CX extract at a dose of 6 mg/kg BW; CX-2: CX extract at a dose of 30 mg/kg BW. SD is not displayed in this table.

**Table 4.** The increase in concentration level (ICL) of *R*- and *S*-WF from the 7th to 9th day in all groups of rats after CX extract administering concomitantly in the multiple-dose study.

Group	Day-7		Day-9		ICL (%)	
	*R*-WF	*S*-WF	*R*-WF	*S*-WF	*R*-WF	*S*-WF
Control	2.02	4.50	2.04	4.54	1.04	0.93
CX-1	2.28	5.47	2.33	5.77	2.02	5.47
CX-2	3.44	6.85	3.98	8.68	15.68*	26.64*
FZ	4.87	8.90	NA	NA	NA	NA

Control: 2% PGA; FZ: Fluconazole at a dose of 6 mg/kg BW. CX-1: CX extract at a dose of 6 mg/kg BW; CX-2: CX extract at a dose of 30 mg/kg BW; ICL: increase in concentration level from 7th to 9th-day observation. NA: not available. sample discontinuing because of major bleeding or died. SD is not displayed in this table.

*The difference is statistically significant at a level of *p* < 0.05 (by Kruskall-Wallis method).

## Discussion

Warfarin has narrow therapeutic index and high intersubject variability, therefore its treatment management needs to be carefully performed by considering some factors contributing to alteration in both pharmacodynamic and pharmacokinetic responses. Furthermore, drug-herbs interaction is one of the important factors that alter PK parameters and contribute to the WF dose adjustment. Note that the pharmacokinetic alteration tends to be caused by induction or inhibition of metabolizing enzymes such as cytochrome P450 family 3 subfamily A4 (CYP3A4), CYP2C9, and CYP2C19. However, the pharmacodynamic response is altered by some vegetables or herb with high vitamin K content when concomitantly consumed with WF. In this study, the influence of one of the famous herbs in Indonesia called *temulawak* or Javanese turmeric (*Curcuma xanthorrhiza*) on the pharmacokinetics of WF was assessed. FZ was used as a drug model for the positive control which has clearly inhibited CYP2C9 activity, hence it increased the WF concentration level. Several studies have reported the possible interaction between warfarin and Fluconazole (Gericke 1993; Black et al. 1996; Kunze et al. 1996). FZ is a strong inhibitor of CYP2C9 which is the main enzyme in metabolizing the more potent form of warfarin (*S*-WF), to become an inactive product such as *S*-(6) and *S*-(7)-hydroxywarfarin. It is also a potent inhibitor for the formation of *R*-(10)-hydroxywarfarin that is often catalysed by CYP3A4 (Kunze et al. 1996). Another study proved that the *S*-WF concentration significantly increased after concomitant administration with 400 mg/day fluconazole for 6 days in human volunteers. The formation of *S*-(6) and *S*-(7)-hydroxywarfarin was inhibited by approximately 70%, while *R*-(10) and other metabolites were by 45–88% (Black et al. 1996). Therefore, FZ decisively is applicable as a model to validate a correct method for studying the effect of certain compounds on the PK of warfarin.

The present study also confirmed that the WF plasma concentration profile in rats treated by fluconazole was significantly higher than in the control group as seen in Table 1, Figures 1, and 2. This result indicates that FZ is a very good substance to be used for a positive control in assessment of the drug/food/herbs effects on the pharmacokinetics alteration of warfarin. As many have reported that the mechanism of FZ in increasing warfarin concentration is by inhibiting the activity of the CYP2C9 enzyme. Therefore, this study can be considered to have a good validation for assessing the effect of other substances on the pharmacokinetics of warfarin, in this case we propose to study how the effects of *Curcuma xanthorrhiza* (which is often used by most Indonesians people for maintaining health) on the pharmacokinetic parameters of warfarin.

Regarding the effect of CX extract on warfarin, this study reported that almost all PK parameters of *R*- and *S*-WF in the CX-1 were not significantly different from those in the control group (Table 2) meaning that the extract of *Curcuma xanthorriza* in the usual dose did not affect WF pharmacokinetics. But, most of PK parameters in the CX-2 were significantly altered from those in the control (Table 2). This result indicates that the extract of *Curcuma xanthorrhiza* in the dose of 3 time higher than usual dose affected the elimination of WF to become longer in the rats. This could be explained from the value of k, t½ and C_L_ parameters. The k parameter of *R*- and *S*-WF was almost two-times lower in the CX-2 compared to control group (0.043 vs. 0.065 h^–1^ for *R*-WF and 0.026 vs. 0.042 h^−1^ for *S*-WF, *p* < 0.05, Kruskal-Wallis) meaning that the WF elimination rate was slower when administered with CX-2. This also corresponds to the value of clearance (C_L_) and elimination half-life (t_1/2_) where the C_L_ of *R*-and *S*-WF were lower than in the control and consequently, the t_1/2_ was also (2.5-fold) higher in the CX-2 compared to the control meaning that the WF was stay longer in the body rats. Therefore, the co-administration of CX extract at a higher dose could make *R*-and *S*-WF stay longer in the body.

The most important PK parameter that represents the bioavailability of a drug is the AUC_0-inf_ (total systemic exposure). In this study we can state that CX extract at the higher dose could increase the bioavailability of *R*- and *S*-WF (77.54 vs. 35.27 mg.h/L, *p* < 0.05 for *R*-WF and 316.26 vs. 40.16 mg.h/L, *p* < 0.01 for *S*-WF, Kruskal-Wallis). These results indicate that co-administration of CX extract in the 3-times higher dose than usual could significantly increase the systemic exposure of WF which could lead to the supratherapeutic or bleeding in the body of rats. Over all, from the single-dose study on rats after concomitant administration, the CX extract influenced the pharmacokinetics of WF in a high dose but not in the usual dose. Just as many animal studies have large variabilities in PK parameters, our data also showed large variabilities in PK parameters. On the other hand, our data showed a considerable difference in AUC. This indicated that the clinical effect can be very large, suggesting that the drug dose can fluctuate about 5-fold. However, no statistically significant difference was obtained in the PK parameters due to the variation in CX-1 group, on the other hand, the value of AUC was more than doubled to control. This suggests that the dose in the steady state may differ by about 2 times, and it is considered that detailed clinical evaluation is desired in the future.

From the multiple-dose study, the profile of *R*- and *S*-WF plasma concentration curve showed that the CX-2 line was higher than the control group, but not the CX-1, meaning that CX-2 extract could increase the level of WF concentration. To make a deep analysis, we also assessed the increase in concentration level of *R*- and *S*-WF (ICL) from the 7th to 9th day as listed in Table 4. The ICL was moderately higher at 15 and 25% in the CX-2, but no significant increase in the CX-1 and the control group (less than 6%). This result indicates that the administration CX-2 could increase the level of WF steady state concentration (C_ss_) on the body rats. In this case, we had taken the blood sampling at the point of C_max_(ss) indicating that the upper range of WF therapeutic index might be exceeded so that it is needed for further clinical evaluation of this interaction.

Generally, this study showed that administration of CX extract concomitantly on rats has the ability to alter the pharmacokinetics of *R*- and *S*-WF particularly at a high dose (3–5 times the usual). Further investigation of the chemical interaction between CX extract and CYP2C9 enzymes is suggested to further explore the mechanism of pharmacokinetic alteration.

In this study, we focus on the PK parameters of WF and pointed out the possibility of interaction between CX and WF. We have not evaluated the effect of CX on the pharmacodynamic effects of WF, and no report about pharmacodynamic effects of CX on WF was found at least for this point. If CX also affects the pharmacodynamic effects of WF, clinically greater interactions can occur. So, to assess the risk of concomitant use of CX and WF, it will be necessary to analyse in detail from the perspectives of both pharmacokinetic and pharmacodynamic interactions in clinical evaluations in the future.

## Conclusions

Based on the single and multiple-dose studies, the concomitant administration of C*urcuma xanthorrhiza* extract at a normal daily dose did not significantly alter the pharmacokinetic profiles and parameters of warfarin but it tends to do that when high (more than 3 times the usual). This study demonstrated the need for clinical evaluation of the interaction between CX and WF.
